# Nailfold Capillaroscopy With USB Digital Microscopy in Connective Tissue Diseases: A Comparative Study of 245 Patients and Healthy Controls

**DOI:** 10.3389/fmed.2021.683900

**Published:** 2021-08-06

**Authors:** Kumutnart Chanprapaph, Wuttidej Fakprapai, Preeyachat Limtong, Poonkiat Suchonwanit

**Affiliations:** Division of Dermatology, Faculty of Medicine, Ramathibodi Hospital, Mahidol University, Bangkok, Thailand

**Keywords:** capillaroscopy, dermoscopy, nailfold, systemic lupus erythematosus, dermatomyositis, systemic sclerosis

## Abstract

**Background:** Nailfold capillaroscopy (NFC) is a valuable tool to detect microcirculation abnormalities in connective tissue diseases (CTDs). However, whether the universal serial bus (USB) digital microscopy used as onychoscopy is as effective as the videocapillaroscopy in determining the diagnostic and prognostic values of CTDs remains to be determined.

**Objective:** This study aims to investigate NFC features of systemic lupus erythematosus (SLE), dermatomyositis (DM), and systemic sclerosis (SSc) patients and compare with normal controls as well as examine which feature could differentiate among CTDs. Furthermore, we aim to explore different capillaroscopic abnormalities and their association with disease activity.

**Methods:** Nailfold images were taken from patients and healthy controls using a USB digital microscopy. Patterns on the capillary morphology, diameter, architecture, and density were recorded and compared. We further determined the NFC findings in SLE, DM, and SSc and corresponded to their respective disease activity scoring system.

**Results:** A total of 245 participants, consisting of 54 SLE, 32 DM, and 51 SSc patients, as well as 108 controls, were enrolled. All capillaroscopic features, except for tortuous capillaries, were significantly more common in CTDs than healthy control (all *p* < 0.05). A multinomial logistic regression analysis revealed that bushy capillaries had significantly higher odds for both SLE and DM than SSc (OR: 4.10, 95% confidence interval (CI): 1.71–9.81, *p* = 0.002 and OR: 7.82, 95% CI, 2.86–21.38, *p* < 0.001, respectively). Elongated capillaries demonstrated significant odds for SLE compared with SSc (OR: 3.35, 95% CI: 1.005–11.20, *p* = 0.049), while prominent subpapillary plexus showed greater odds for SLE compared with both DM and SSc (OR: 2.75, 95% CI: 1.07–7.02, *p* = 0.03 and OR: 5.78, 95% CI: 2.29–14.58, *p* < 0.001, respectively). The presence of hemorrhage, enlarged capillaries, and the low-density index had significantly higher odds in favor of SSc than SLE. Bushy capillaries were the only pattern with a strong association for DM over SSc. The presence of enlarged capillaries indicated higher SLE severity, but no specific finding was related to DM or SSc skin scores.

**Conclusions:** Nailfold capillaroscopic examination using a digital microscope is a valuable method for the diagnosis of SLE, DM, and SSc. Several morphologic patterns can help differentiate among CTDs; however, the prognostic significance of this method requires further investigations.

## Introduction

Nailfold capillaroscopy (NFC) represents the best method to analyze microvascular abnormalities among connective tissue diseases (CTDs) since capillaroscopic changes of the nailfold have been well established in many CTDs. It is commonly used for differentiating primary from secondary Raynaud's phenomenon and for the diagnosis of scleroderma-spectrum disorders, including systemic sclerosis (SSc), dermatomyositis (DM), undifferentiated CTD, and mixed CTD (1). NFC changes can also be detected in other chronic autoimmune rheumatic diseases, namely systemic lupus erythematosus (SLE), Sjogren's syndrome, rheumatoid arthritis, and antiphospholipid syndrome (2–5).

SSc is a prototype of scleroderma-spectrum disorders which exhibits the typical scleroderma pattern through NFC assessments. Characteristic capillaroscopic changes of scleroderma contain enlarged capillaries, bushy capillaries, capillary hemorrhage, disorganization of the normal distribution of capillaries, and avascular area (6). At present, nailfold microvascular abnormalities are of great importance both at the time of diagnosis and during follow-up and have been established as a part of the most recent classification criteria for the diagnosis of SSc, the American College of Rheumatology and European League Against Rheumatism (ACR/EULAR) 2013 for SSc criteria (7).

Capillaroscopic alterations were described in patients with inflammatory myopathies, typically exhibiting scleroderma-capillary patterns. DM patients appear to have more frequent nailfold capillary abnormality than polymyositis. Giant capillaries and bushy capillaries with other characteristics in the scleroderma pattern were found in 74% of cases (8). Conversely, NFC changes in SLE have been documented to be less specific (2, 9). The most frequent capillaroscopic features of SLE are tortuous capillaries, elongated capillaries, and prominent subpapillary plexus (10, 11).

Although NFC is extremely useful for detecting various nailfold abnormalities, being the gold standard to discover microvascular changes in the nailfold, it requires a special equipment, the videocapillaroscopy, which is relatively expensive, time consuming, and not widely available in most healthcare settings. Videocapillaroscopy is the current gold standard for NFC assessment (12). Dermoscopy is an easily applicable, low-cost, noninvasive diagnostic technique generally used among dermatologists to evaluate inflammatory and pigmentary skin conditions. There are broadly two types of dermoscopy: (i) hand-held dermoscopy, allowing 10–20 times magnification and (ii) videodermoscopy, providing higher magnification up to 1,000 times (13). Hair and scalp dermoscopy, also known as trichoscopy, has become a standard evaluation for alopecia and other scalp disorders (14). Currently, trichoscopic features among CTDs have been well described and may indicate their diagnostic and prognostic values (15–19). Onychoscopy refers to the examination of the nail unit using dermoscopy. It has been further adapted for the assessment of nailfold vascular abnormalities. There is growing evidence suggesting that microvascular nailfold changes reflect the presence of certain autoantibodies and the disease activity in patients with CTDs (8, 11, 20–29). However, whether the NFC changes possess prognostic values in rheumatic disorders remains to be determined.

Currently, reports on nailfold capillaroscopic features using a digital microscope as an onychoscopy in CTDs are limited, and direct comparison among the most common rheumatologic disorders, namely, SLE, DM, and SSc are sparse. This study aimed to explore the differences among onychoscopic microvascular features in SLE, DM, SSc, and healthy controls. The secondary objective was to determine NFC findings and their association to the disease activity.

## Materials and Methods

This cross-sectional, case-control study was approved by the Mahidol University Institutional Review Board (MURA 2019/263) and conducted at Ramathibodi Hospital, Bangkok, Thailand from February 2018 to February 2019. Informed consent to participate in the study was obtained from SLE, DM, and SSc patients, as well as healthy controls. SLE individuals who fulfilled the Systemic Lupus International Collaborating Clinics (SLICC) 2012 criteria and/or American College of Rheumatology (ACR) 1997 criteria (30), DM cases that met the Bohan and Peter criteria and/or the European League Against Rheumatism/American College of Rheumatology (EULAR/ACR) IIM criteria (31), and patients with SSc who satisfied the ACR/EULAR 2013 SSc criteria and/or the ACR 1980 criteria were recruited from the rheumatology and/or dermatology connective tissue disease clinics (7). The diagnosis and assessment of disease activity were made by a rheumatology-dermatology specialist. For healthy controls, healthcare personnel and patients who presented at the dermatology outpatient department for minor or cosmetic consultations, except for conditions involving the nails, and did not report signs and symptoms of CTD were recruited. Patients with other CTDs such as overlapping CTD, rheumatoid arthritis, Sjogren's syndrome, mixed CTD, and undifferentiated CTD were excluded from the study. Other exclusion criteria were patients who had traumatized finger and vascular occlusive diseases.

Nailfold examination was performed by a universal serial bus (USB) digital microscope with a polarizing light (Dinolite® AnMo Electronics Corporation, Hsinchu, Taiwan) on both 4th and 5th fingers with × 200 magnification because their capillaries are the most visible. All examinations were operated by the same investigator. Patients were acclimatized for a minimum of 15–20 min before the examination at a room temperature (20–25°C). A drop of immersion oil was applied to the nailfold skin to improve resolution (32). Examination of the first distal row of capillaries was done to assess nailfold capillaroscopic features. Four consecutive images of each finger were captured. A blinded evaluation of images was conducted by an expert dermatologist.

**Figure 1:**
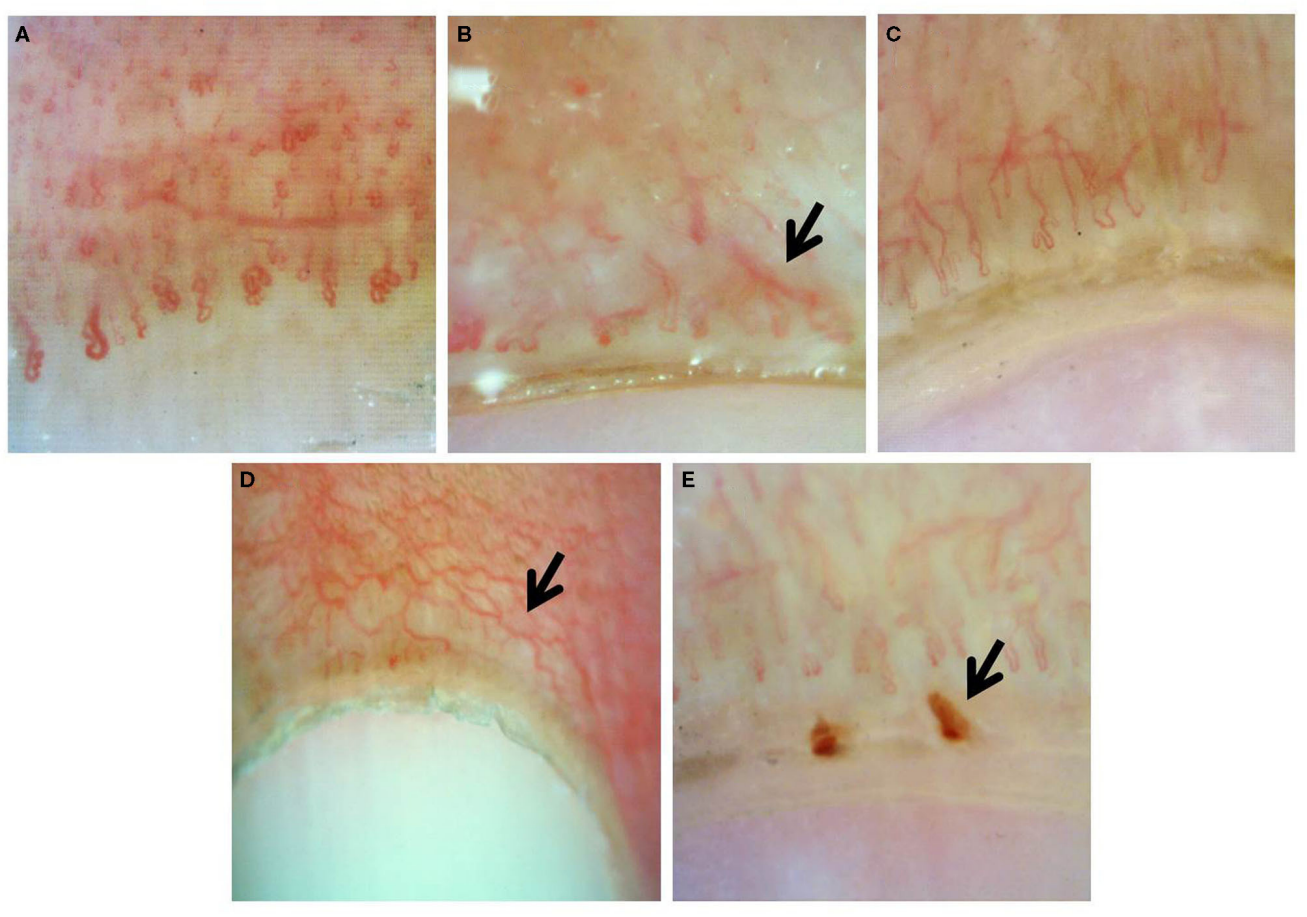
Onychoscopic morphologic features: **(A)** tortuous capillaries, **(B)** bushy capillaries, **(C)** elongated capillaries, **(D)** prominent subpapillary plexus, and **(E)** capillary hemorrhage.

**Figure 2:**
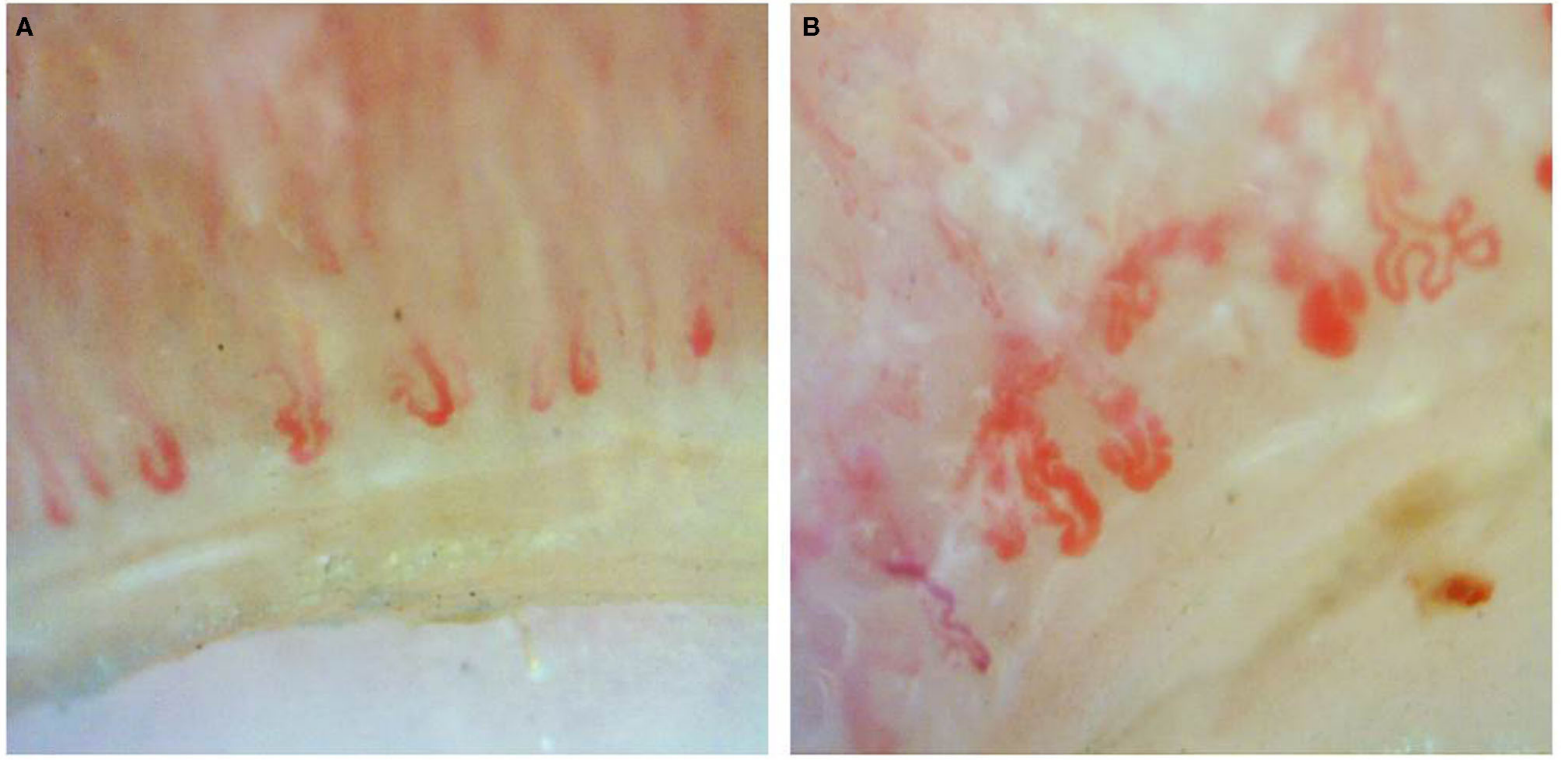
Diameter index of onychoscopy: **(A)** enlarged capillaries and **(B)** giant capillaries.

**Figure 3:**
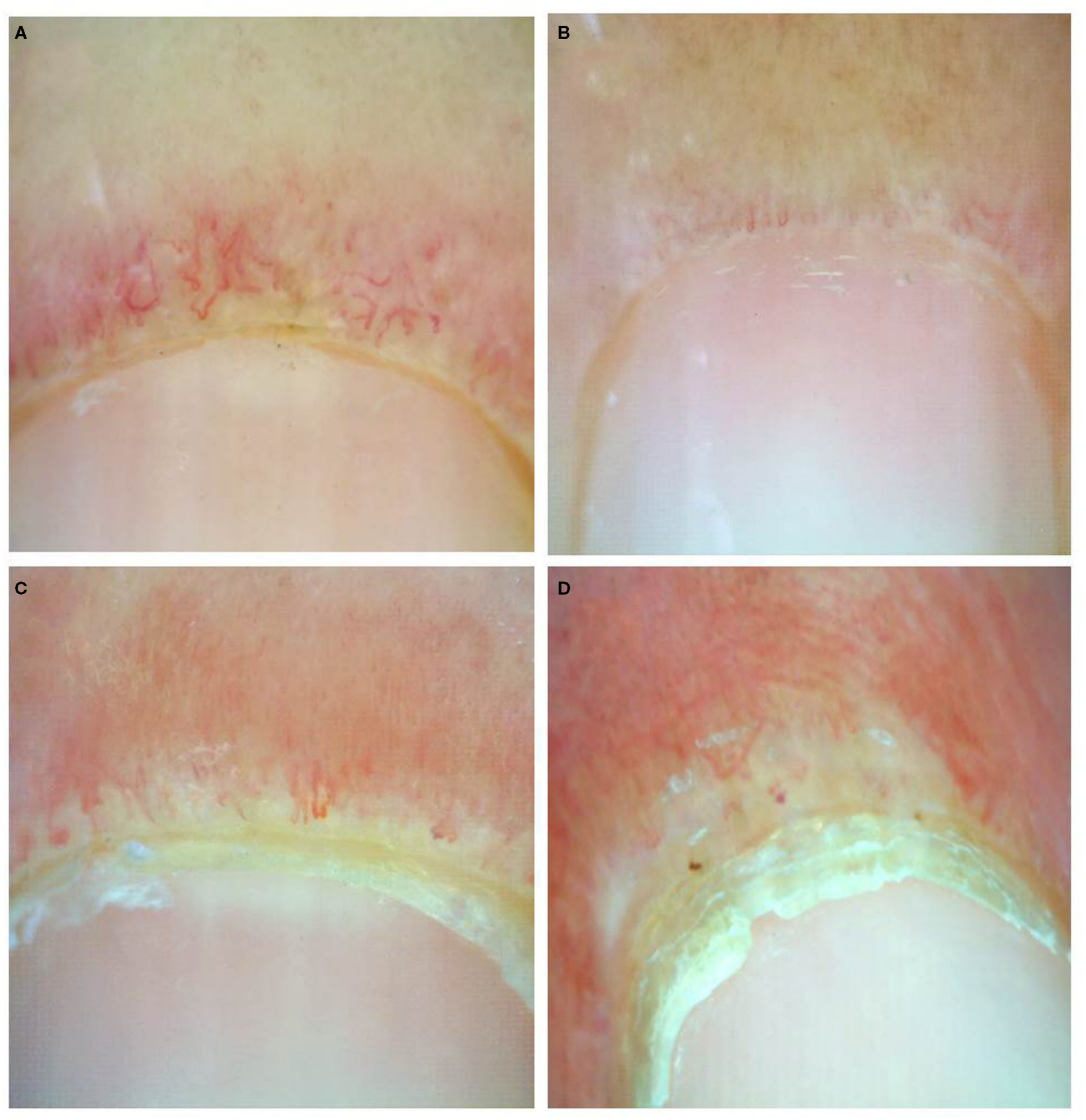
Onychoscopic architectural features: **(A)** disorganized capillaries, **(B)** avascular area grade 1, **(C)** grade 2, and **(D)** grade 3.

**Figure 4:**
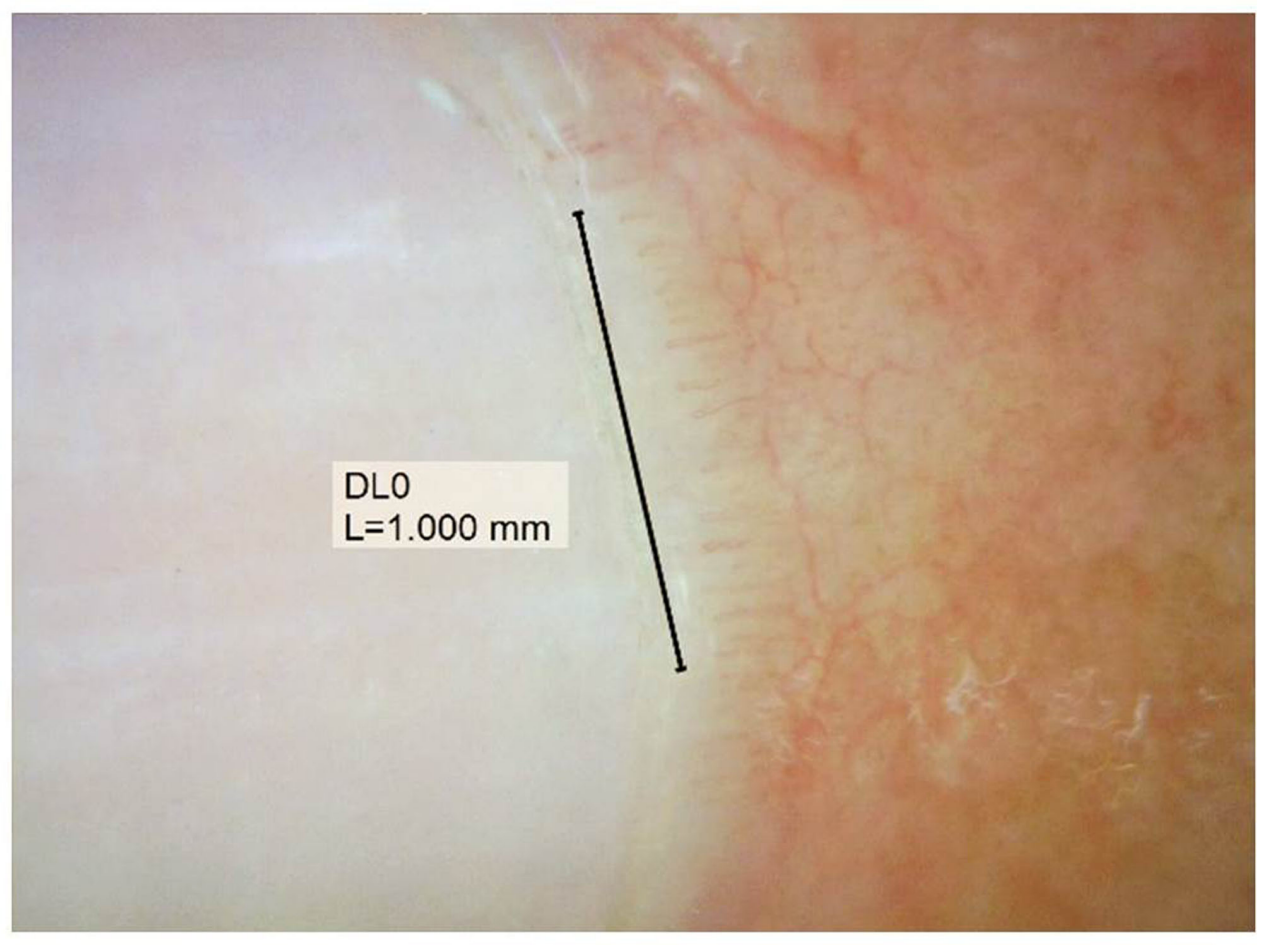
Assessment of capillary density, counting the visible end row capillaries under a 90° angle.

Documented onychoscopic microvascular features consisted of morphology, diameter, architecture, and density of the nailfold capillaries. Morphologic parameters included tortuous capillaries (curled, crossed, or meandering capillaries; Figure 1A) (32), bushy capillaries (one capillary with three buddings; Figure 1B) (33), elongated capillaries (capillary loops longer than 300 μm; Figure 1C) (9, 34, 35), subpapillary plexus (vascular network at the base of the finger; Figure 1D) (34), and hemorrhages (extracapillary brown aggregations of erythrocytes and dived into punctate and confluent hemorrhages; Figure 1E) (9, 32, 35). The diameter index consisted of enlarged capillaries (capillaries with a diameter of arterial or venous limb wider than 20 μm; Figure 2A) (36), and giant capillaries (capillaries with a diameter wider than 50 μm; Figure 2B) (9, 32, 34). Architectural features included disorganized capillaries (irregular capillary pattern; Figure 3A) (34) and avascular area (distinct area in the nailfold where two or more adjacent capillaries are missing; Figures 3B–D) (34). Furthermore, the avascular area was quantitatively classified as grade 0 (no avascular area), grade 1 (mild or one or two avascular areas per millimeter), grade 2 (moderate or more than two avascular areas per millimeter), and grade 3 (severe or large and confluent avascular area) (35, 37). Capillary density was defined as the number of capillary loops in a 1-mm span of the distal row (9, 34, 37). We obtained capillary density by counting only the visible end row capillaries under a 90° angle. A capillary loop was considered a distal loop if the angle between the apex of that capillary and the apex of its two adjacent capillaries is >90° (Figure 4) (38). Any of the aforementioned features found at least in one finger was considered positive for capillary abnormality.

Nailfold capillaroscopic features among the three CTDs and controls were compared. Additionally, the correlation between onychoscopic microvascular changes and disease activity was additionally evaluated. The disease activity in SLE was assessed by using the Systemic Lupus Erythematosus Disease Activity Index 2000 (SLEDAI-2K) (39). We also evaluated the NFC changes in association to the skin scores of DM and SSC by using the Cutaneous Assessment tool for Myositis (CAT activity score) and modified Rodnan skin score, respectively (40, 41).

Data were analyzed using Stata 14.0 (Stata Corp LLC, College Station, TX). Results were expressed as mean ± standard deviation (SD) for continuous data and percentage for categorical data. Statistical analysis employed the chi-squared test or Fisher's exact test for categorical data and one-way ANOVA or Kruskal-Wallis test for continuous data. Multinomial logistic regression was used to determine the correlation among the three CTDs. A *p*-value of <0.05 was considered statistically significant. The magnitude of association was presented as odds ratio (OR) accounting for a 95% confidence interval (CI).

## Results

Of 255 subjects included in the study, 10 were excluded due to the diagnosis of overlapping CTD (*n* = 5), inadequate data to assess disease severity (*n* = 3), and having traumatized fingers (*n* = 2). The remaining patients for SLE, DM, and SSc groups were 54, 32, and 51, respectively. For healthy controls, 108 subjects were enrolled. Table 1 demonstrates demographic data in patients with SLE, DM, SSc, and healthy controls. The mean age of the SLE cases was younger than DM and SSc patients (34.9, 52.2, and 56.8, respectively). Female predominance was observed in patients with CTDs. All patients and healthy controls had Fitzpatrick skin type III-V and were of Southeast Asian ethnicity. Raynaud's phenomenon was reported in 5 (9.3%), 4 (12.5%), and 29 (56.9%) of SLE, DM, and SSc patients, respectively. Anti U1-ribonucleoprotein (U1RNP) antibody was present in 14/35 (40%) of SLE, 0/22 (0%) of DM, and 3/30 (10%) of SSc cases. Patients manifesting Raynaud's phenomenon and/or anti-U1RNP antibody were found in 15 (40.5%), 4 (16.7%), and 29 (69%) of SLE, DM, and SSc groups, respectively.

**Table 1:** Comparison of demographic data of patients with connective tissue diseases and healthy controls.

**Variable**	**SLE (** ***N*** **= 54)**	**DM (** ***N*** **= 32)**	**SSc (** ***N*** **= 51)**	**Controls (** ***N*** **= 108)**
Mean age (year ± SD)	34.9 ± 15.5	52.2 ± 16.9	56.8 ± 12.5	35.2 ± 10.4
Sex				
• Male	6 (11.1%)	8 (25%)	9 (17%)	41 (37.9%)
• Female	48 (88.9%)	24 (75%)	42 (83%)	67 (62.1%)
• F/M ratio	8:1	3:1	4.6:1	1.6:1
Mean disease duration (year ± SD)	7.6 ± 8.9	3.8 ± 4.5	7.2 ± 6.0	–
Fitzpatrick skin type	III–V	III–V	III–V	III–V
Southeast asian descent	54 (100%)	32 (100%)	51 (100%)	108 (100%)

*DM, dermatomyositis; F, female; M, male; SD, standard deviation; SLE, systemic lupus erythematosus; SSc, systemic sclerosis*.

The comparison between onychoscopic microvascular features in patients with CTDs versus healthy controls revealed that all features, except for tortuous capillaries, were found more in CTDs with statistical significance (all *p* < 0.05) (Table 2). A comparison of NFC changes in SLE, DM, and SSc demonstrated that morphologic patterns consisting of bushy capillaries, subpapillary plexus, hemorrhage, and enlarged capillaries were significantly different among the three conditions. Bushy capillary pattern favored SLE and DM over SSc (*p* < 0.001), whereas elongated capillaries and subpapillary plexus were prominent features of SLE (*p* = 0.003 and *p* < 0.001, respectively). Hemorrhage and enlarged capillaries were also predominant in SSc (*p* < 0.001). The dilated capillaries and avascular areas were seen more in SSc patients, which was statistically greater than SLE and DM cases (*p* = 0.01 and *p* = 0.004, respectively). A quantitative analysis on the degree of avascular areas revealed that the majority of SLE cases had no or mild (72.2%), DM patients had mild to moderate (78.2%), while SSc cases had moderate to severe (64.7%) avascular areas (Supplementary Table 1).

**Table 2:** Comparison of onychoscopic features between connective tissue diseases and healthy controls.

**Onychoscopic features**	**SLE (** ***N*** **= 54)**	**DM (** ***N*** **= 32)**	**SSc (** ***N*** **= 51)**	***p*** **-Value (among CTDs)**	**Controls (** ***N*** **= 108)**	***p*** **-Value (CTDs vs. controls)**
Tortous capillaries *N* (%)	48 (88.8%)	31 (96.8%)	50 (98%)	0.125	96 (88.8%)	0.125
Bushy capillaries *N* (%)	27 (50%)	21 (65.6%)	10 (19.6%)	<0.001^*^	4 (3.7%)	<0.001^*^
Elongated capillaries *N* (%)	12 (22.2%)	3 (9.3%)	4 (7.8%)	0.073	4 (3.7%)	0.003^*^
Subpapillary plexus *N* (%)	28 (51.8%)	9 (28.1%)	8 (15.6%)	<0.001^*^	15 (13.8%)	<0.001^*^
Hemorrhage *N* (%)	10 (18.5%)	11 (34.4%)	26 (50.9%)	0.002^*^	3 (2.7%)	<0.001^*^
Enlarged capillaries *N* (%)	25 (46.2%)	21 (65.6%)	38 (74.5%)	0.01^*^	27 (25%)	<0.001^*^
Disorganized capillaries *N* (%)	43 (79.6%)	31 (96.8%)	46 (90.1%)	0.05	21 (19.4%)	<0.001^*^
Avascular areas *N* (%)	41 (75.9%)	30 (93.7%)	49 (96%)	0.004^*^	10 (9.2%)	<0.001^*^
Density (mean ± SD)	6.83 ± 1.95	5.31 ± 1.86	5.09 ± 1.69	0.592	7.43 ± 1.20	<0.001^*^

*CTDs, connective tissue diseases; DM, dermatomyositis; SD, standard deviation; SLE, systemic lupus erythematosus; SSc, systemic sclerosis*.

* *Statistically significant*.

A multinomial logistic regression analysis comparing nailfold capillaroscopic findings among the three CTDs is demonstrated in Table 3. The results on morphologic pattern revealed that the presence of bushy capillaries had significantly higher odds for having both SLE and DM than SSc (OR: 4.10, 95% CI: 1.71–9.81; *p* = 0.002 and OR: 7.82, 95% CI: 2.86–21.38; *p* < 0.001, respectively). Elongated capillaries demonstrated significant odds for SLE compared with SSc (OR: 3.35, 95% CI: 1.005–11.20; *p* = 0.049), while prominent subpapillary plexus showed greater odds for SLE compared with both DM and SSc (OR: 2.75, 95% CI: 1.07–7.02; *p* = 0.03 and OR: 5.78, 95% CI: 2.29–14.58; *p* < 0.001, respectively). For other morphological and architectural patterns (i.e., hemorrhage, enlarged capillaries, and avascular area) as well as the low-density index, significantly higher odds were in favor of SSc than SLE. Bushy capillaries were the only pattern that showed a strong association for DM over SSc (OR: 7.82, 95% CI: 2.86–21.38; *p* < 0.001). We further performed quantitative analysis on the degree of avascular areas. The absence of avascular area revealed higher odds for SLE over SSc, while extensive avascular area (grades 2–3) had significantly greater odds for SSc and DM (Supplementary Table 2).

**Table 3:** Multinomial logistic regression analysis of onychoscopic features in SLE, DM, and SSc patients.

**Onychoscopic features**	**SLE vs. DM**	**SLE vs. SSc**	**DM vs. SSc**
Tortous capillaries	OR: 0.25	OR: 0.16	OR: 0.62
	*p*-Value: 0.22	*p*-Value: 0.095	*p*-Value: 0.73
	95% CI: 0.02–2.24	95% CI: 0.01–1.37	95% CI: 0.03–10.27
Bushy capillaries	OR: 0.52	OR: 4.10	OR: 7.82
	*p*-Value: 0.16	*p*-Value: 0.002^*^	*p*-Value: <0.001^*^
	95% CI: 0.21–1.29	95% CI: 1.71–9.81	95% CI: 2.86–21.38
Elongated capillaries	OR: 2.76	OR: 3.35	OR: 1.21
	*p*-Value: 0.14	*p*-value: 0.049^*^	*p*-Value: 0.80
	95% CI: 0.71–10.66	95% CI: 1.005–11.20	95% CI: 0.25–5.82
Subpapillary plexus	OR: 2.75	OR: 5.78	OR: 2.10
	*p*-Value: 0.03^*^	*p*-Value: <0.001^*^	*p*-Value: 0.17
	95% CI: 1.07–7.02	95% CI: 2.29–14.58	95% CI: 0.71–6.18
Hemorrhage	OR: 0.43	OR: 0.21	OR: 0.50
	*p*-Value: 0.10	*p*-Value: 0.001^*^	*p*-Value: 0.14
	95% CI: 0.15–1.18	95% CI: 0.09–0.52	95% CI: 0.20–1.25
Enlarged capillaries	OR: 0.45	OR: 0.29	OR: 0.65
	*p*-Value: 0.08	p-Value: 0.004^*^	*p*-Value: 0.38
	95% CI: 0.18–1.11	95% CI: 0.12–0.67	95% CI: 0.24–1.71
Disorganized capillaries	OR: 0.12	OR: 0.42	OR: 3.36
	*p*-Value: 0.053	*p*-Value: 0.14	*p*-Value: 0.27
	95% CI: 0.01–1.02	95% CI: 0.13–1.32	95% CI: 0.37–30.25
Avascular areas	OR: 0.21	OR: 0.12	OR: 0.61
	*p*-Value: 0.05	*p*-Value: 0.009^*^	*p*-Value: 0.63
	95% CI: 0.04–1.00	95% CI: 0.02–0.60	95% CI: 0.08–4.57
Density	OR: 1.21	OR: 1.74	OR: 0.22
	*p*-Value: 0.09	*p*-value: <0.001^*^	*p*-Value: 0.52
	95% CI: 0.91–2.42	95% CI: 1.12–2.35	95% CI: −0.48 to 0.93

*DM, dermatomyositis; OR, odds ratio; SLE, systemic lupus erythematosus; SSc, systemic sclerosis; 95% CI, 95% confidence interval*.

* *Statistically significant*.

The correlation between NFC findings and disease activity of CTDs is shown in Table 4. The presence of enlarged capillaries was significantly associated with higher median SLEDAI scores in SLE patients (*p* = 0.01). However, no specific feature was related to DM and SSc cutaneous changes using the CAT score and modified Rodnan skin score, respectively.

**Table 4:** Onychoscopic findings in relations to disease activity in SLE, DM, and SSc patients.

**Onychoscopic features**	**SLE**			**DM**			**SSc**		
	**Median SLEDAI score in patients with the features (IQR)**	**Median SLEDAI score in patients without the features (IQR)**	***p*** **-Value**	**Median CAT score in patients with the features (IQR)**	**Median CAT score in patients without the features (IQR)**	***p*** **-Value**	**Median modified Rodnan score in patients with the features (IQR)**	**Median modified Rodnan score in patients without the features (IQR)**	***p*** **-Value**
Tortuous capillaries	3.5 (0–8)	10 (5–14)	0.03^*^	3 (2–5)	0 (0–0)	0.08	4 (1–12)	10 (10)	0.42
Bushy capillaries	4 (1–14)	4 (0–6)	0.18	3 (2–5)	3 (2–5)	0.82	7.5 (2–13)	3 (1–10)	0.27
Elongated capillaries	4 (1–11.5)	4 (1–8)	0.68	4 (2–5)	3 (2–5)	0.64	3 (0–10.5)	5 (2–11)	0.41
Subpapillary plexus	4 (0–9)	4 (2–9)	0.80	3 (2–5)	3 (1–5)	0.44	2 (0.5–7)	6 (2–12.5)	0.21
Hemorrhage	4.5 (2–10)	4 (0.5–8.5)	0.52	3 (2–5)	4 (2–5)	0.84	4 (1–8)	6 (2–13)	0.60
Enlarged capillaries	7 (2–14)	2 (0–6)	0.01^*^	4 (2–5)	2 (1–4)	0.11	5 (2–12.5)	4.5 (0.5–9)	0.50
Disorganized capillaries	4 (1–11)	2 (0–6)	0.23	3 (2–5)	0 (0–0)	0.08	5 (1–11)	6 (2–11.5)	0.69
Avascular areas	4 (2–10)	2 (0–6)	0.07	3 (2–5)	2 (0–4)	0.34	6 (2–12)	1 (0–2)	0.15
Decrease density	4.5 (0–13)	3 (1.5–6)	0.24	3 (2–5)	3 (1–4.5)	0.62	5 (2–12)	3.5 (0–7)	0.43

*CAT, cutaneous assessment tool for myositis; DM, dermatomyositis; IQR, interquartile range; SLE, systemic lupus erythematosus; SLEDAI-2K, Systemic Lupus Erythematosus Disease Activity Index 2000; SSc, systemic sclerosis*.

* *Statistically significant*.

## Discussion

Nailfold capillaroscopic examination using a USB digital microscopy has been demonstrated to provide high-quality images and comparable ability to differentiate between patients with SSc and healthy controls in previous studies with a small number of subjects (12, 42, 43). The method offers a cheaper and more readily available device comparing with videocapillaroscopy. Our study on a relatively high number of patients demonstrates the significant value of a USB digital microscope used as onychoscopy for the detection of nailfold capillary abnormalities among the three most common CTDs, namely SLE, DM, and SSc. To the best of our knowledge, this is the first study to compare NFC abnormalities among CTDs and the normal population with this method. Although information regarding the comparison of NFC features in patients with autoimmune CTDs vs. healthy controls has been previously reported in Caucasian and Japanese individuals (44, 45), there is no data in the Southeast Asian population. We confirm that numerous onychoscopic microvascular parameters in the morphologic indexes (i.e., bushy capillaries, elongated capillaries, subpapillary plexus, and hemorrhage), diameter patterns (i.e., enlarged capillaries), architectural forms (i.e., disorganized and avascular pattern), and decreased capillary density were distinct features for CTDs and rarely seen in normal individuals.

Previous reports have documented an increase in tortuosity as one of the important NFC findings in CTDs, particularly for SLE patients (9, 10). Our results have suggested otherwise, tortuous capillary morphology was a fairly common finding in healthy controls and should not be considered a valuable index to identify CTDs. The scleroderma pattern consists of two or more of the following features, enlarged capillaries, bushy capillaries, capillary hemorrhage, disorganized capillaries, and avascular area, presented in at least two nailfolds (6). These characteristic microvascular changes are well recognized for SSc. DM is considered a scleroderma-spectrum disorder; therefore, the scleroderma pattern is also commonly observed in DM patients (8, 46, 47). In the present study, we confirm that the majority (over 90%) of SSc and DM patients possessed NFC features of the scleroderma-type pattern. In multinomial logistic regression analysis, the scleroderma-type features on onychoscopy, namely, hemorrhage, enlarged capillary, and avascular area were not suggestive of SSc over DM or vice versa. However, only the bushy capillary morphology revealed significantly higher odds for DM (OR: 7.82, 95% CI: 2.86–21.38; *p* < 0.001). Therefore, we postulate that although DM is considered a scleroderma-spectrum disorder, possessing similar nailfold capillaroscopic features to SSc, bushy capillary formation is a valuable NFC finding that favored DM over SSc.

Evidence to date has shown that capillary nailfold changes in SLE patients present a smaller percentage of the scleroderma-type pattern and hold fewer specific characteristics (9, 10, 27–29, 39, 45, 48, 49). However, our results were discordant with previous reports. Of 51 SLE patients, 79.6%, 75.9%, 50%, 46.2%, and 18.5% had disorganized architecture, avascular areas, bushy capillaries, enlarged capillaries, and hemorrhage as their NFC findings, respectively. Therefore, using the aforementioned findings, over three-fourths of our SLE patients would fulfill the definition of the scleroderma pattern of NFC. In line with several previous reports, the higher prevalence of scleroderma-type pattern in SLE patients may be in part due to a relatively high number of SLE patients (40.5%) possessing Raynaud's phenomenon and/or anti-U1RNP antibody (50, 51). It would be informative to further explore whether or not scleroderma-type pattern on nail fold of SLE reflexes an SLE subset with subclinical features of SSc such as Raynaud's phenomenon. As our data showed that the scleroderma-type pattern may not invariably differentiate scleroderma-spectrum disorders from SLE, we suggest that individual microvascular nailfold changes and their severity should be considered rather than focusing on a specific pattern spectrum. SLE-type capillary nailfold pattern has been mentioned earlier consisting of tortuous, meandering capillaries, increase diameter, and prominent subpapillary plexus (9, 11, 28, 49). Our study results were concordant with the literature, but with regards to only the elongated capillary and prominent subpapillary plexus being more common in SLE. We also demonstrated that bushy capillary, elongated capillary, and prominent subpapillary plexus were patterns possessing significantly higher odds for SLE compared with SSc patients, while the prominent subpapillary plexus was the sole NFC finding that significantly favored SLE over DM. Moreover, in quantitative analysis for the degree of avascular areas, the absence of avascular area revealed significant odds for SLE over SSc, while extensive avascular areas favored both SSc and DM over SLE.

Microvascular changes play a central role in the pathophysiology of SLE, DM, and SSc. The similarities and dissimilarities on the NFC features may reflect their microvascular pathology. Endothelial injury is thought to be the initial vascular insult in SSc. Endothelial cell apoptosis, vasoconstriction, and vasculopathy are pronounced in SSc. A vast amount of tissue hypoxia in SSc patients may parallel the predominant nailfold avascular areas and the decrease capillary density seen on onychoscopy. Angiogenesis consequently occurs as compensation to chronic hypoxia, but the process is defective and dysregulated in SSc patients. An endothelial activation factor, namely vascular endothelial growth factor (VEGF), is highly expressed and has shown to be associated with decreased nailfold capillary density in patients with SSc (52).

VEGF is considered a key regulator of angiogenesis for SLE and DM as well. Kuryliszyn-Moskal et al. demonstrated that endothelial activation markers, namely VEGF, endothelin-1, E-selectin, and thrombomodulin were associated with increased capillaroscopic changes in SLE. Moreover, patients with moderate to severe capillaroscopic abnormalities (e.g., elongated, tortuous, meandering, ramified, and/or disarranged capillaries) showed higher levels of VEGF (23). In DM, microvascular injury is mediated by the humeral process with secondary vascular depletion, hypoxia, and myofiber necrosis. Subsequently, local angiogenesis occurs as compensation for vascular reduction. Angiogenic factors such as VEGF and angiogenin may play a role in the pathogenesis of DM and correspond to their onycoscopic changes (53). Therefore, similar to SSc, onychoscopic findings in DM may display both ischemia (e.g., avascular area and disorganized capillaries) and aberrant angiogenesis (e.g., enlarged capillaries and bushy capillaries). Nevertheless, further studies to determine the true pathomechanism and biomarkers that reflect the microvascular changes in CTDs are warranted.

The correlation of NFC alterations with the disease activity in SLE, DM, and SSc has been reported with variable results. In the present study, SLE patients with higher SLEDAI scores were associated with the presence of enlarged capillaries on onychoscopy than those without. Our results were in line with the literature showing higher disease activity defined by the SLEDAI, Systemic Lupus Activity Measures, and the European Consensus Activity Measures in SLE patients with NFC changes (11, 23, 27, 29). Documents on the microvascular nailfold abnormalities and disease activity in DM and SSc have been rather inconsistent. In DM and/or polymyositis, while several studies have shown a significant correlation between capillary damage with disease activity (e.g., myositis damage index, myositis disease activity assessment tools, and the global disease activity), many others have not (8, 54–59). Reports on the relationship between NFC and disease severity in SSc have been quite controversial, ranging from significant predictive association to no association. The significant heterogeneity reported are due to the differences in disease duration, specific organ complication (e.g., pulmonary vascular/parenchymal disease, myocardial dysfunction, and cutaneous sclerosis), scoring systems, and the parameter used in NFC (20, 60–64). In the present study, we found no correlation between nailfold capillaroscopic patterns with DM and SSc skin activity score, using the CAT score and modified Rodnan skin score, respectively. Further studies with validated disease severity/outcome parameters, analysis technique, and scoring system are much needed to determine the relationship between NFC features and the disease severity and organ-specific complications among CTDs.

The present study is subjected to several limitations. We did not determine NFC abnormalities of other chronic rheumatic disorders such as Sjogren's syndrome, rheumatoid arthritis, and antiphospholipid syndrome. Additional limitations were its cross-sectional, single-center study nature, with population restricted to Southeast Asians, and lack of prospective monitoring in order to evaluate alterations of NFC features and their relationship with disease activity over time. Our designated severity measures could not reflect the severity of disease in all aspects. Finally, being conducted in a tertiary care center means the study was prone to referral bias.

## Conclusion

Our study confirms that a USB digital microscope used as onychoscopy is a valuable tool to detect nailfold capillary changes in SLE, DM, and SSc patients. Several morphologic patterns and their severity can help differentiate among CTDs. Therefore, the device may serve as a relevant diagnostic instrument and could be implemented in clinical practice. However, determining the prognostic significance of onychoscopy requires a large cohort study with validating assessment on disease severity and organ-specific complication.

## Data Availability Statement

The raw data supporting the conclusions of this article will be made available by the authors, without undue reservation.

## Ethics Statement

The studies involving human participants were reviewed and approved by the Mahidol University Institutional Review Board for Ethics in Human Research. The patients/participants provided their written informed consent to participate in this study.

## Author Contributions

PS: Conceptualization, writing-review and editing. PS, KC, and WF: methodology. PS and PL: validation. KC and WF: formal analysis PS, KC, WF, and PL: investigation. KC and WF: data curation and writing-original draft preparation. All authors have read and agreed to the published version of the manuscript.

## Conflict of Interest

The authors declare that the research was conducted in the absence of any commercial or financial relationships that could be construed as a potential conflict of interest.

## Publisher's Note

All claims expressed in this article are solely those of the authors and do not necessarily represent those of their affiliated organizations, or those of the publisher, the editors and the reviewers. Any product that may be evaluated in this article, or claim that may be made by its manufacturer, is not guaranteed or endorsed by the publisher.
