# Supplementary material for: Nailfold Capillaroscopy With USB Digital Microscopy in Connective Tissue Diseases: A Comparative Study of 245 Patients and Healthy Controls
Source: Front Med (Lausanne). 2021 Aug 6;8:683900. doi: 10.3389/fmed.2021.683900 (PMC8377356; doi:10.3389/fmed.2021.683900)
Supplement: Supplementary file 1 [file Data_Sheet_1.PDF]

Supplementary Table 1. Comparison of quantitative measurement of avascular area among SLE, DM, and SSc patients

**Onychoscopic finding, N (%)**	**SLE (N = 54)**	**DM (N = 32)**	**SSc (N = 51)**	**P value**
**Avascular area**				
• **Grade 0**	13 (24%)	2 (6.2%)	2 (3.9%)	**0.004***
• **Grade 1**	26 (48.2%)	10 (31.3%)	16 (31.4%)	0.14
• **Grade 2**	10 (18.5%)	15 (46.9%)	18 (35.3%)	**0.01***
• **Grade 3**	5 (9.3%)	5 (15.6%)	15 (29.4%)	**0.02***

DM, Dermatomyositis; SLE, Systemic lupus erythematosus; SSc, Systemic sclerosis. *Statistically significant.

Supplementary Table 2. Multinomial logistic regression analysis of quantitative measurement among SLE, DM, and SSc patients

Variable	OR	95%CI	P value
**No avascular area (Grade 0)**			
• DM vs. SSc	1.63	0.21-12.21	0.63
• SLE vs. SSc	7.76	1.65-36.43	**0.009***
• SLE vs. DM	4.75	0.99-22.66	0.05
**Extensive avascular area (Grade 2-3)**			
• DM vs. SSc	0.90	0.36-2.27	0.83
• SLE vs. SSc	0.20	0.09-0.47	**< 0.001***
• SLE vs. DM	0.23	0.09-0.58	**0.002***

DM, Dermatomyositis; OR, odds ratio; SLE, Systemic lupus erythematosus; SSc, Systemic sclerosis; 95%CI, 95% confidence interval. *Statistically significant.

This is a provisional file, not the final typeset article
